# Serum discrimination and phenotype assessment of coronary artery disease patents with and without type 2 diabetes prior to coronary artery bypass graft surgery

**DOI:** 10.1371/journal.pone.0234539

**Published:** 2020-08-05

**Authors:** James R. Hocker, Megan Lerner, Stan A. Lightfoot, Marvin D. Peyton, Jess L. Thompson, Subrato Deb, Mathew Reinersman, R. Jane Hanas, Russel G. Postier, Barish H. Edil, Harold M. Burkhart, Jay S. Hanas

**Affiliations:** 1 Department of Biochemistry and Molecular Biology The University of Oklahoma Health Sciences Center, Oklahoma City, Oklahoma, United States of America; 2 Department of Surgery The University of Oklahoma Health Sciences Center, Oklahoma City, Oklahoma, United States of America; 3 Department of Medicine The University of Oklahoma Health Sciences Center, Oklahoma City, Oklahoma, United States of America; Case Western Reserve University School of Medicine, UNITED STATES

## Abstract

Diabetes Mellitus (DM) accelerates coronary artery disease (CAD) and atherosclerosis, the causes of most heart attacks. The biomolecules involved in these inter-related disease processes are not well understood. This study analyzes biomolecules in the sera of patients with CAD, with and without type (T) 2DM, who are about to undergo coronary artery bypass graft (CABG) surgery. The goal is to develop methodology to help identify and monitor CAD patients with and without T2DM, in order to better understand these phenotypes and to glean relationships through analysis of serum biomolecules. Aorta, fat, muscle, and vein tissues from CAD T2DM patients display diabetic-related histologic changes (e.g., lipid accumulation, fibrosis, loss of cellularity) when compared to non-diabetic CAD patients. The patient discriminatory methodology utilized is serum biomolecule mass profiling. This mass spectrometry (MS) approach is able to distinguish the sera of a group of CAD patients from controls (*p* value 10^−15^), with the CAD group containing both T2DM and non-diabetic patients. This result indicates the T2DM phenotype does not interfere appreciably with the CAD determination versus control individuals. Sera from a group of T2DM CAD patients however are distinguishable from non-T2DM CAD patients (p value 10^−8^), indicating it may be possible to examine the T2DM phenotype within the CAD disease state with this MS methodology. The same serum samples used in the CAD T2DM versus non-T2DM binary group comparison were subjected to MS/MS peptide structure analysis to help identify potential biochemical and phenotypic changes associated with CAD and T2DM. Such peptide/protein identifications could lead to improved understanding of underlying mechanisms, additional biomarkers for discriminating and monitoring these disease conditions, and potential therapeutic targets. Bioinformatics/systems biology analysis of the peptide/protein changes associated with CAD and T2DM suggested cell pathways/systems affected include atherosclerosis, DM, fibrosis, lipogenesis, loss of cellularity (apoptosis), and inflammation.

## Introduction

Type 2 Diabetes Mellitus (T2DM) has increased in recent decades to epidemic proportions, in large part due to increases in obesity-inducing diets and adoption of sedentary life styles [1, 2]. T2DM is a significant risk factor for enhanced development of cardiovascular disease (CVD), coronary artery disease (CAD), and atherosclerosis, resulting in increased probabilities of dying from cardiovascular events compared with non-diabetics [3]. The disease atherosclerosis is in part an inflammatory disorder at sites of endothelial tissue injury in arterial walls [4]. Low-density lipoproteins and monocytes have roles in forming fatty deposits at sclerotic sites [5]. Molecular mechanisms of diabetes-accelerated CAD and atherosclerosis are not well understood, although insulin and lipid dysregulation as well as hyperglycemia are purported to have prominent roles [2]. Improved understanding of underlying biochemical mechanisms of these and other cardio-pathological processes should lead to better monitoring of disease states, identification of important cellular pathways affected and potential therapeutic targets, and novel biomarkers for monitoring these disease states and their treatments. Analysis of peripheral blood and blood products (plasma and serum) for biomarkers is one productive/possible avenue toward understanding CAD and associated co-morbidities like DM. Such approaches as hypothesized in this study will provide clues to understanding how DM accelerates CAD and atherosclerosis. In addition, the possibility exists of discovering novel biomarkers to predict CAD and atherosclerosis risk, and actual presence and progression in DM patients.

There are a number of existing protein biomarkers in the peripheral blood used for CVD, CAD, and T2DM risk analysis. These include acute phase proteins and pro-inflammatory markers C-reactive protein (CRP), fibrinogen, plasminogen activator inhibitor (PAI)-1, lipoprotein associated phospholipase A_2_ ((Lp-PLA 2), and interleukin (IL)-6 [6]. However, definitive progress on such molecular analyses of disease phenotypes has been slow. At present, most risk assessment for development and progression of CVD, CAD, and diabetes includes analyses of anthropometrics, lifestyle and socioeconomics, metabolics, psychosocial stress, and environmental pollution exposure [6]. The purpose of the present study is to introduce and test a novel methodology to possibly better identify/monitor patients with CAD and related T2DM. In addition, this methodology lends itself to gleaning underlying biochemical mechanisms as well as identifying potential novel biomarkers and therapeutic targets. This methodology involves an all-liquid mass spectrometry (MS) platform approach using unfractionated serum analysis to distinguish CVD patients from healthy controls and from CAD patients with and without T2DM.

This mass spectrometry (MS) serum platform approach was successful in identifying patients with early-stage cancers, neurological infections, and traumatic brain injury (TBI) and related concussion disorders [7–10]. The specific disease monitoring and discriminating ability of this MS platform is likely due to the large number of distinguishing components analyzed at the same time [9–11]. The more differing components so analyzed the greater the disease discriminatory powers of a biomarker platform. The major hypothesis of this approach is that disease conditions such as CAD and T2DM elicit multiple disease-specific and systemic biochemical responses from organs and tissues. Disease-specific biomolecules will be shed or secreted into the peripheral blood which are observable and distinguishable with this serum electro-spray ionization mass spectrometry (ESI-MS) mass profiling platform. [12, 13].

These physiological changes can take the form of defense and homeostatic responses, stress responses, and direct inputs from disease tissues [10, 14, 15]. Peptides and proteins possibly identified in such studies as this one, distinguishing CAD patients with T2DM versus CAD alone, could provide clues about mechanisms and potential therapeutic targets as well as novel biomarkers for these disease states. The ability of this platform to distinguish/monitor the sera of patients with CAD or CAD plus T2DM, based on their respective serum biomolecule mass peak profiles, is demonstrated for the patient groups examined in this study. In addition, Bioinformatics/systems biology analysis of the peptide/protein changes associated with CAD and T2DM suggest cell pathways/systems affected include fibrosis, lipogenesis, oxidative stress, loss of cellularity (apoptosis), inflammation, and cardiomyopathy.

## Materials and methods

### Study participants

This cross-sectional retrospective study was conducted among patient and control volunteers from the University of Oklahoma Health Sciences Center (OUHSC) in Oklahoma City. The study was approved by the OUHSC Human Studies Institutional Review Board (IRB#16199 & #1268). A written informed consent for study participation was obtained for all volunteers. Study participants were enrolled and provided blood samples (before any treatments) for the project in calendar year 2013. The designation of T2DM was indicated in the patient’s medical records. Volunteers consisted of one group of patients (N sample size of 25) diagnosed with CAD with and without T2DM, and another group composed of N = 25 control individuals. Sixty four percent of the CAD volunteer group had T2DM. CABG patients and controls ranged in age from 42 to 79 and 35 to 70, respectively.

### Serum collection and tissue histology

Sera were obtained from patient peripheral blood from an arm vein at the University of Oklahoma associated Hospitals according to standard procedures [14]. Sera aliquots (250 μl) were frozen at -80 ^o^C, and not reused after initial freezing and thawing. Patient-related information is listed in Table 1 in Results. For hematoxylin-eosin staining (H&E), tissues were fixed in 10% neutral buffered formalin, dehydrated, and embedded in paraffin. Sections were de-paraffinized, rehydrated, and stained as exhibited previously [15].

**Table 1:** Demographic characteristics of eligible study groups.

Group	Ethnic	N	Patient age (years)			BMI Mean	Tobacco History	ETOH	COPD	T2DM	CABG	CABG+T2DM
		(F:M)	Group Mean (F:M)	F: Mean (range)	M: Mean (range)	Group (F: M)	N (F: M)	N (F: M)	N (F: M)	N (F: M)	N (F: M)	N (F: M)
**CABG**	**All patients**	25 (15–10)	60 (63.16–55.48)	63.6 (52–79)	54 (42–60)	30.91 (32.6–30.15)	16 (8–8)	7 (4–3)	6 (5–1)	16 (12–4)	9 (3–6)	16 (12–4)
**Controls**	**All patients**	25 (17–8)	57.2 (59.88–45.72)	59.9 (52–65)	51.5 (35–70)	27.69 (27.1–29.36)	9 (5–4)	17 (11–6)	0 (0)	0 (0)	0 (0)	0 (0)
**CABG**	**White**	18 (11–7)	60.17 (63.82–54.43)	63.8 (53–79)	54.4 (42–60)	30.41 (30.88–29.66)	12 (6–6)	6 (4–2)	6 (5–1)	11 (8–3)	0 (0)	11 (8–3)
**Controls**	**White**	24 (17–7)	58 (59.88–53.43)	59.9 (52–65)	53.4 (35–70)	27.59 (27.1–28.71)	9 (5–4)	16 (11–5)	0 (0)	0 (0)	0 (0)	0 (0)
**CABG**	**African American**	6 (4–2)	60 (62.5–55)	62.5 (52–71)	55 (54–56)	32.02 (34.33–27.4)	4 (2–2)	0 (0)	0 (0–0)	5 (4–1)	1 (0–1)	5 (4–1)
**Controls**	**African American**	0 (0)	(na-na)	na	na	na	0 (0)	0 (0)	0 (0)	0 (0)	0 (0)	0 (0)
**CABG**	**Hispanic**	1 (0–1)	57 (na-57)	na	57 (57)	33.4 (na-33.4)	0 (0)	1 (0–1)	0 (0)	0 (0)	1 (0–1)	0 (0)
**Controls**	**Hispanic**	1 (0–1)	38 (na-38)	na	38 (38)	30 (na-30)	0 (0)	1 (0–1)	0 (0–0)	0 (0)	0 (0)	0 (0)

N (Number of patients); F (Female patients); M (Male patients); na (not applicable or none available); CABG (Cardiac Artery Bypass Graft); BMI (Body Mass Index); ETOH (Ethyl Alcohol); COPD (Chronic Obstructive Pulmonary Disease); T2DM (Type 2 Diabetes Mellitus).

### Electrospray mass spectrometry of sera from CAD patients and controls

The ADVANTAGE LCQ ion-trap electrospray MS instrument (ThermoFisher), was used for “leave one out [serum sample] cross validation” (LOOCV) analysis of serum MS spectra and for tandem MS/MS peptide/protein structural identifications. Full-range calibration of the LCQ was performed following recommended manufacturer protocols. All HPLC grade solvents were purchased from ThermoFisher. Each patient’s sera (4 μl) was individually analysed after dilution of 1 to 300 into a solution of 50% methanol and 2% formic acid, and separated into 3 aliquots. The samples were directly infused by loop injection (20 μl) into the nano-source of the mass spectrometer fitted with a 20 micron inner diameter (100 micron outer diameter) fused silica (Polymicro Technologies) tip. Solvent flow was at a rate of 0.5 μl/min using an Eldex MicroPro series 1000 pumping system and with previously described instrument settings [8, 9]. Patient sera were analysed randomly through acquisition of high-resolution triplicate mass spectra. The spectra were sampled at an m/Z (mass divided by charge) resolution of two hundredths over an m/Z range of 400 to 2000 and positive ion spectra were averaged over a period of 20 minutes for each injection. Each patient’s spectral data was extracted using the manufacturer's software (Qual Browser: version 1.4SR1) as “Nominal Mass Spectra” (whole unit intensity spectral data). Data were locally scaled (normalized) to a sum value of 100 intensity in non-overlapping segments of 10 m/Z for the entirety of the spectrum. MS spectral peak assignments and areas were calculated as centroid m/Z peak area values (valley to valley) using Mariner Data Explorer 4.0.0.1 software (Applied BioSystems). Centroid area is defined as the area of the peak calculated from its geometric m/Z center.

To obtain information on peptide/protein changes taking place among patients, tandem MS/MS mass peak peptide/protein structure identifications were performed with the Advantage LCQ ion-trap instrument in similar fashion as described previously [7, 8, 10]. 108 unit-Dalton m/Z ions encompassing the m/Z range of 900 to 1008 were analysed for nine CAGB patient sera samples and nine CABG plus T2DM patient sera samples. This particular range represents a median range of about 100 m/Z units between the 700 to 1200 range that previously provided serum MS/MS peptide identification data. 35% fragmentation ionization energy was utilized for each peak, and each parent ion m/Z was isolated, fragmented, and observed for 5 minutes. Analysis of MS/MS signals was performed using ThermoFisher Proteome Discoverer 1.0 sp1 on human and *T*. *solium* non-redundant databases downloaded from National Center for Biotechnology Information (NCBI), 02/01/2016. Serum samples on average contained 1.95 (range: 0–5) parent ions with significant differences of standard MS spectral data between the pre and post MS/MS scans of the 108 parental ions analyzed. MS/MS search-related settings: [enzyme name = no-enzyme (no digest)], precursor mass tolerance = 1.8 Da, fragment mass tolerance = 0.8 Da, b & y ions were scored, and dynamic modifications were noted for oxidation (C, M amino acids), phosphorylation (S, T, Y), methylation (C), all with maximum of 4 modifications per peptide.

Peptide/protein identifications required a minimum of 2 unique peptides and a cross correlation range (Xcorr) minimum of 1.7, in line with previous studies [10, 16]. Identified sequences were searched using Basic Local Alignment Search Tool (BLAST) against NCBI human and non-human specific *T*. *solium* non-redundant databases to retrieve current gene notation for analysis. A “hit” in the database search is scored for a MS/MS scan when the Xcorr, identifying a peptide sequence, is higher than the minimum cut off. Multiple scans identifying the same peptide or protein related sequence would be identified as multiple “hits”. For Ingenuity Pathway Analysis (IPA, QIAGEN), identified gene names and the number of Identified MS/MS sequence “hits” were imported each as log_2_ ratios of CABG/CABG+T2DM [17]. Imported proteins were manually inspected and verified for protein function using Medline/PubMed.

### Statistical and quantitative analysis

Mass spectral data were exported into Excel in a format providing rounded unit m/Z and intensity values from the raw data files and locally normalized/scaled to a value of 100 for the m/Z sum for all values in segments of 10 m/Z from 400–2000. MS spectral peak assignments were calculated as centroid m/Z peak area values (valley to valley). Leave one [serum sample] out cross validation (LOOCV) was used to distinguish serum samples between binary groupings CABG patients vs CABG patients with T2DM vs controls. LOOCV is one procedure to reduce over-fitting of large datasets [18–20]. The triplicate averaged serum spectra mass peak areas between groups were analysed for significant differences at individual m/Z values using Student’s *t* -tests (one-tailed, unequal variance, significance designated at *p* < 0.05), leaving out a different sample (e.g., CABG or control) in succession to build each unique N– 1 LOOCV “left in” significant mass peak dataset. All significant peaks utilized for these separations were at least 0.3% of the normalized maximum peak area. The mass peaks of each “left out” sample are then compared, peak area to peak area, to all the “left in” mass peaks in their unique N-1 LOOCV dataset. This comparison involves the use of a peak classification value (PCV) metric at each significant “left in” peak of the LOOCV dataset. Whether a “left out” peak area falls above or below this midpoint metric determines its classification. For example, in Fig 3 panel B peak 836 is classified as a “CABG” peak in the “left in” database. If the 836 peak from the “left out” sample has a peak area above the PCV then it is classified as a “CABG”. If it falls below or equal to this PCV then the “left out” peak is classified as “Control”. Such peak classifications are performed for all “left out” peaks in all “left out” serum samples against their respective N-1 “left in” LOOCV mass peak databases. This procedure can result in patient sera having less than 100% peak adherence to one group, resulting in serum having both a percentage of “CABG peaks” and “Control peaks”. These % of total mass peaks classified (e.g., as CABG) for the left-in dataset is assigned each “left out” sample and plotted on the y axis vs the individual serum samples on the x-axis in Fig 4 panel A. To check for over-fitting of large datasets, random grouping of serum samples from subject groups being compared in binary fashion was obtained using the RAND (randomization) function in Excel and manually balanced to retain gender and age ratios of the initial groups. Upon randomization, the identical mass peak LOOCV analysis was performed as described above and seen in Fig 4 panel B. To obtain potential statistical powers for group sample sizes (ability to detect type II errors-false negatives), Cohen’s *d* effect size values are calculated from the binary group % LOOCV means and standard deviations in Table 2 [21]. Statistical power using given sample sizes is calculated as described [22].

**Table 2:** Binary patient groups: CABG vs control and CABG vs CABG with T2DM comparison test metrics.

**I Test Metrics (group 1 vs. group 2)**	**% LOOCV** **Mean (SD) group 1**	**% LOOCV Mean (SD) group 2**	**True Positive group 1**	**False Positive group 2**	**True Negative group 2**	**False Negative group 1**	**N**	**Figure Number**
**CABG vs. Control**	56.88% (5.25%)	37.77% (4.88%)	30/25 (100%)	1/25 (4%)	19/25 (96%)	0/25 (0%)	CABG (N = 25), Control (N = 25)	3 A,B & 4 A,B
**CABG [training set] vs. Control [training set]**	61.84% (6.20%)	38.27% (5.15%)	19/20 (95%)	0/20 (0%)	30/20 (100%)	1/20 (5%)	CABG [training set] (N = 20) Control [training set] (N = 20)	5 A,B,D
**CABG [blinded samples] vs. Control [blinded samples]**	54.62% (5.49%)	46.21% (3.82%)	5/5 (100%)	1/5 (20%)	4/5 (80%)	0/5 (0%)	CABG [blinded samples] (N = 5) Control [blinded samples] (N = 5)	5 C,D
**CABG with T2DM vs. Control without T2DM**	68.24% (5.69%)	40.41% (4.98%)	15/15 (100%)	0/24 (0%)	24/24 (100%)	0/15 (0%)	CABG without T2DM (N = 15), Control without T2DM (N = 24)	6 A
**CABG without T2DM vs. Control without T2DM**	61.16% (5.55%)	38.86% (4.35%)	9/9 (100%)	0/9 (0%)	9/9 (100%)	0/9 (0%)	CABG without T2DM (N = 9), Control without T2DM (N = 9)	6 B
**CABG without T2DM vs. Control without T2DM**	56.60% (5.69%)	20.52% (5.53%)	15/15 (100%)	0/9 (0%)	9/9 (100%)	0/15 (0%)	CABG without T2DM (N = 15), Control without T2DM (N = 9)	6 C
**CABG with T2DM vs. CABG without T2DM**	68.97% (5.44%)	30.13% (3.96%)	9/9 (100%)	0/9 (0%)	9/9 (100%)	0/15 (0%)	CABG with T2DM (N = 9), CABG without T2DM (N = 9)	6 D
**CABG T2DM vs. CABG no T2DM**	59.28% (5.65%)	22.65% (3.84%)	9/9 (100%)	0/12 (0%)	12/12 (100%)	0/9 (0%)	CABG T2DM (N = 9), CABG no T2DM (N = 12)	NS
**II Test Metrics continued**	**Sensitivity**	**Efficiency/(accuracy)**	**True Positive Rate**	**False Positive Rate**	**Specificity**	***P*-value**	**Cohen’s *d***	**Random database *P*-value**	**ROC (Area)**
**CABG vs. Control**	1.0	0.98	1.0	0.04	0.96	5.17 X 10^*−18*^	*3*.*77*	0.05	0.99
**CABG [training set] vs. Control [training set]**	0.95	0.98	0.95	0.00	100	1.07 X 10^−15^	4.13	0.25	0.99
**CABG [blinded samples] vs. Control [blinded samples]**	1	0.9	1.0	0.20	0.80	8.70 X 10^−3^	1.77	-	1.0
**CABG with T2DM vs. Control without T2DM**	1.0	1.0	1.0	0.00	1.0	2.72 X 10^*−15*^	5.20	0.20	1.0
**CABG without T2DM vs. Control without T2DM**	1.0	1.0	1.0	0.00	1.0	8.3 X 10^*−8*^	4.47	0.11	1.0
**CABG without T2DM vs. Control without T2DM**	1.0	1.0	1.0	0.00	1.0	7.96 X 10^*−12*^	6.43	0.21	1.0
**CABG with T2DM vs. CABG without T2DM**	1.0	1.0	1.0	0.00	1.0	1.89 X 10^*−11*^	8.16	0.08	1.0
**CABG T2DM vs. CABG no T2DM**	1.0	1.0	1.0	0.00	1.0	1.74 X 10^*−13*^	7.73	0.36	1.0

Cardiac Arterial Bypass Graft (CABG); Type 2 Diabetes Mellitus (T2DM); Significant peaks utilized: N = 25 & 25, (129–140); m/Z range(400–1908); Training Set: N = 20 & 20, (65–93); m/Z range(434–1291, 1407–1518, 1553–1843, 1904–2000); CABG T2DM vs CABG: (66–73); m/Z range (400–1400); NS (not shown)

### Test metrics

The diagnostic value of a test/procedure is defined by its sensitivity, specificity, predictive value, and efficiency [23, 24]. Test sensitivity was determined from TP/(TP+FN) where TP was the number of true positives for disease presence, and FN was the number of false negatives for disease presence. Specificity was calculated from TN/(TN+FP) where TN is the number of true negatives and FP is the number of false positives. TP, FP, TN, and FN values were determined using “cut off” values between the group means of “% patient serum classified mass peaks” as determined by minus and plus standard deviation (SD])values Fig 4, Fig 5 and Fig 6. Receiver operator characteristic (ROC) curve analysis in Table 2 was performed as described previously [25].

## Results

### Demographic and histological characteristics of study participants

Table 1 exhibits the demographic characteristics of the patient/volunteer subjects in this study. Volunteers consisted of one group of patients (N sample size of 25) diagnosed with CAD with and without T2DM, and another group composed of N = 25 control individuals. By definition, these patients about to undergo the CABG procedure have CAD, and CABG and CAD patient nomenclature will be used inter-changeably in this study. Sixty four percent of the CAD volunteer group (underwent a CABG procedure) had T2DM (16 out of 25). CABG patients and controls ranged in age from 42 to 79 and 35 to 70, respectively. CABG patients are more likely to have T2DM, slightly higher BMIs, and COPD (chronic obstructive pulmonary disease) when compared to control individuals. Both CABG and controls have certain degrees of tobacco and alcohol use.

**Fig 1:**
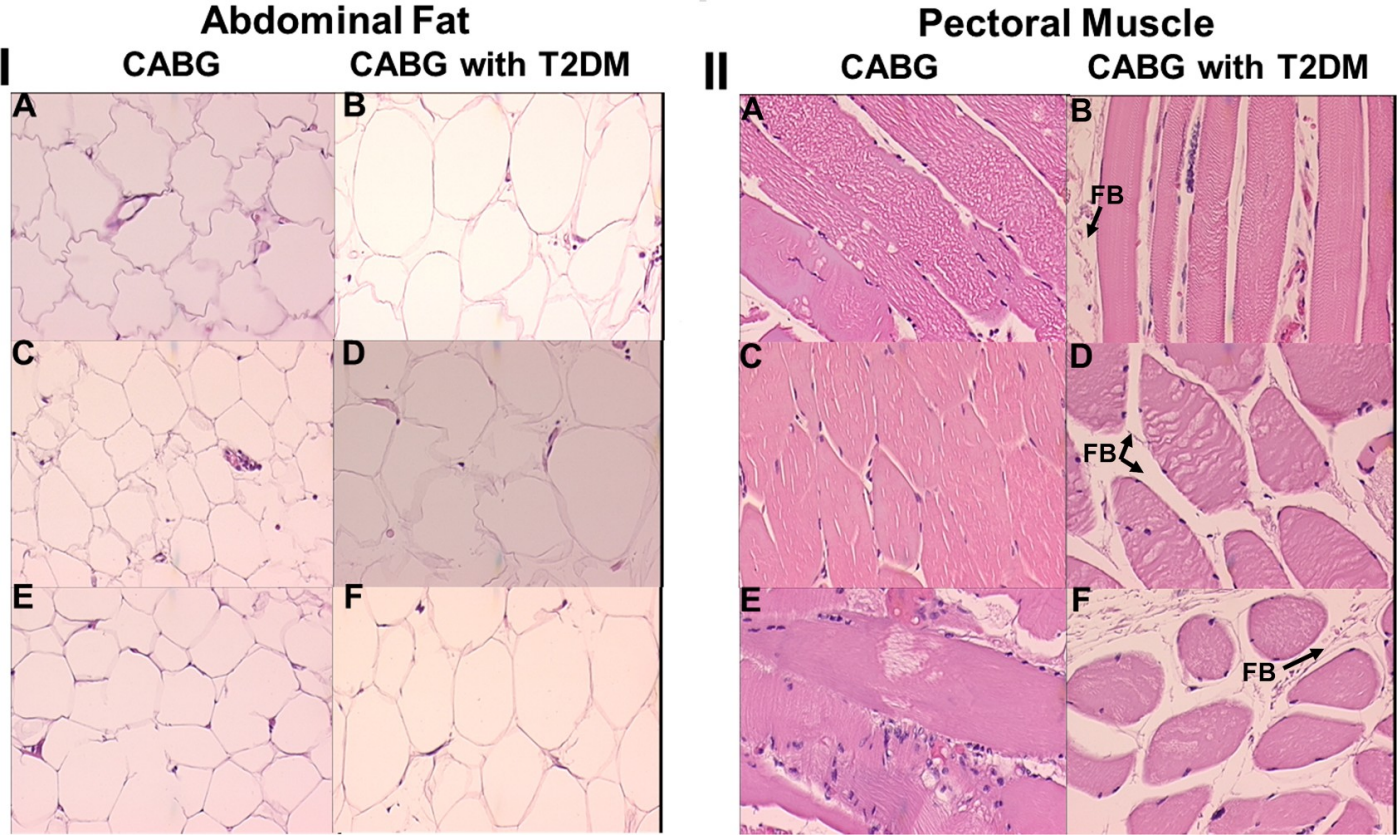
H & E histology of abdominal fat (I) and pectoral muscle (II) from patients with and without T2DM undergoing CABG procedure. The noted feature in panel II is fat build-up (FB). 20x magnification.

**Fig 2:**
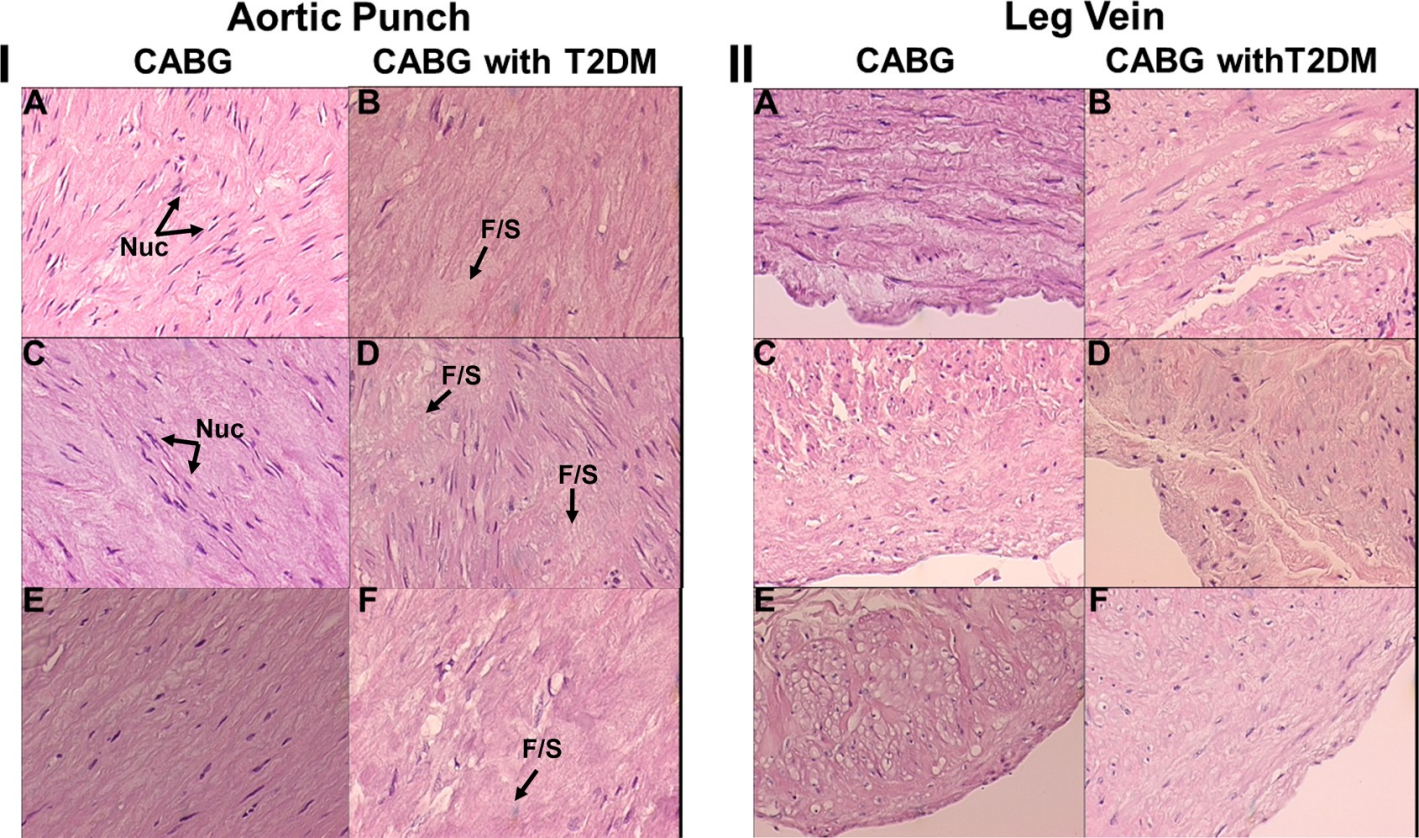
H & E histology of aortic punch (I) and leg vein (II) from patients with and without T2DM undergoing CABG procedure. The noted features in panel I are nuclei (Nuc) and fibrosis/scarring (F/S). 20x magnification.

Tissue histology (thin sections stained with H & E) from CABG patients with or without T2DM is presented in Fig 1 and Fig 2. Fat, muscle, aorta, and vein tissue, obtained at the time of CABG surgery, is illustrated for 3 patients representative of 5, taken from each of the 9 vs 9 binary groups also used in MS/MS range analysis. All the patients exhibited here were obese and all but one was a tobacco smoker. The fat cells from the CABG plus T2DM patients are noticeably larger in Fig 1, panel B, panel D and panel F than in the CABG patients without T2DM in Fig 1 part I panel A, panel C, and panel E. Muscle fibers also have more localized fat between them (FB = fat build up) in the CABG plus T2DM patients Fig 1 part II panel B, Fig 1 part II panel D, and Fig 1 part II panel F. Of interest, the aortas used in the bypass procedure (punch hole tissue) from the CABG patients with T2DM exhibit much more fibrosis (F/S = fibrosis/scarring) and loss of cellularity Fig 2 part I panel B, Fig 2 part I panel D, and Fig 2 part I panel F (apoptosis-loss of dark nuclei, Nuc = nuclei) when compared to CABG patients without T2DM in Fig 2 part II panel A, Fig 2 part II panel C, and Fig 2 part II panel E. Vein tissue between the two groups was deemed similar, within normal limits.

### Distinguishing sera of CAD patients about to undergo CABG procedure from control individuals using ESI-MS LOOCV serum profiling

**Fig 3:**
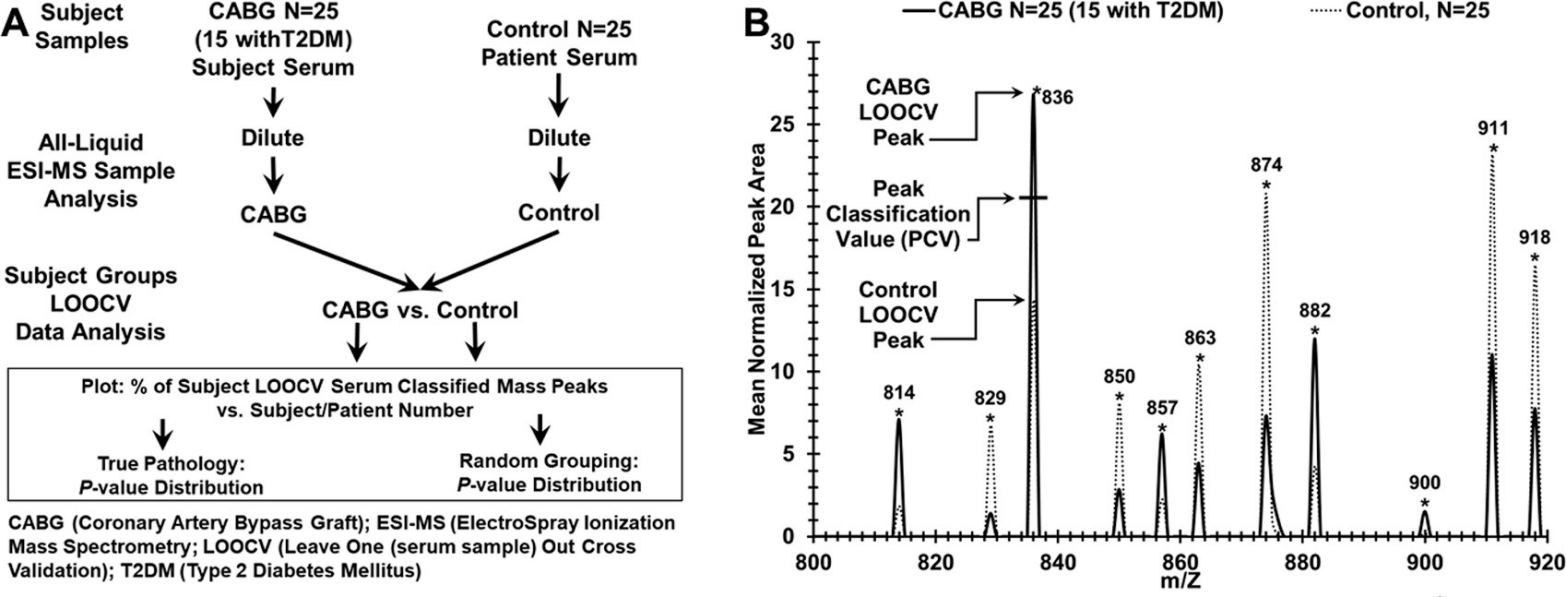
Experimental approach for discriminating sera from patients with CAD before undergoing CABG procedure versus sera from control individuals. (A) Flow chart for serum sample handling and mass spectrometry for binary patient/subject group analysis. Distinguishing control samples from CABG samples are exhibited. (B) Peak Scoring for LOOCV (leave [one serum sample] out cross validation) procedure to classify mass peaks either “CABG” or control from a “left out” sample, over a narrow range (800–920 m/Z is displayed) of significant group discriminatory mass peaks. The PCV (peak classification value) example is exhibited on peak 836 which is used to classify “left out” peaks as either CABG (solid line, peak area above this PCV) or control (dotted line, peak area at or below this PCV).

A hypothesis of the present study is that disease manifestations like CAD and CAD with T2DM can elicit disease-specific biochemical responses in the peripheral blood observable with the serum ESI-MS mass profiling platform described here. Development of such a screening tool would aid in the detection and monitoring of CAD in seemingly healthy individuals as well as in T2DM patients. Fig 3 panel A is a flow diagram depicting mass peak profiling of serum from CABG patients and controls. These binary procedures only require a single sample dilution versus other biomarker platforms which require extensive sample fractionation and handling.

**Fig 4:**
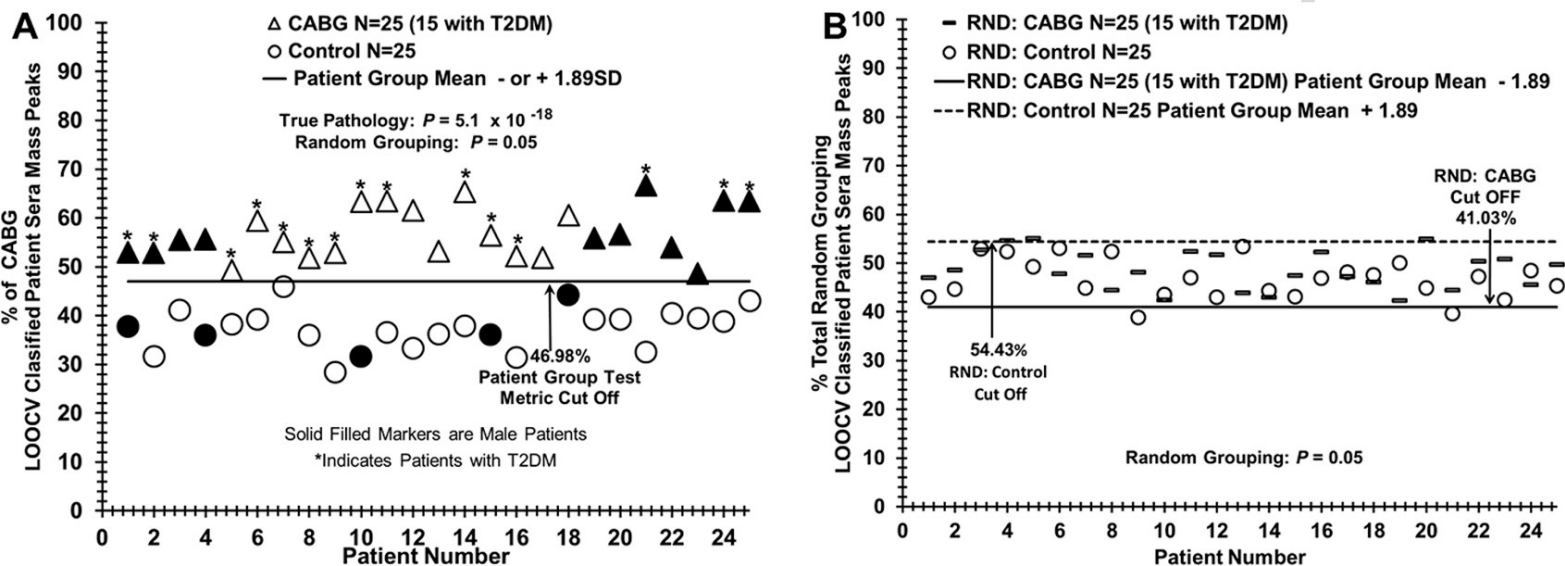
Distinguishing sera from CABG patients versus control individuals using LOOCV mass peak and sample randomization analyses. (A) Serum discrimination of CAD patients before CABG procedure (triangles) from control individuals (circles) by % of LOOCV CABG classified mass peaks. A cut off value is present (- or + SDs [standard deviations] from the CABG or control groups respectively) to determine test metric values (e.g. true positives). Patients with an “*” represent those with T2DM; darkened symbols indicate male subjects. (B) Non-serum sample discrimination when the two different sample groups from (A) are mixed together randomly followed by the same LOOCV mass peak analysis.

**Fig 5:**
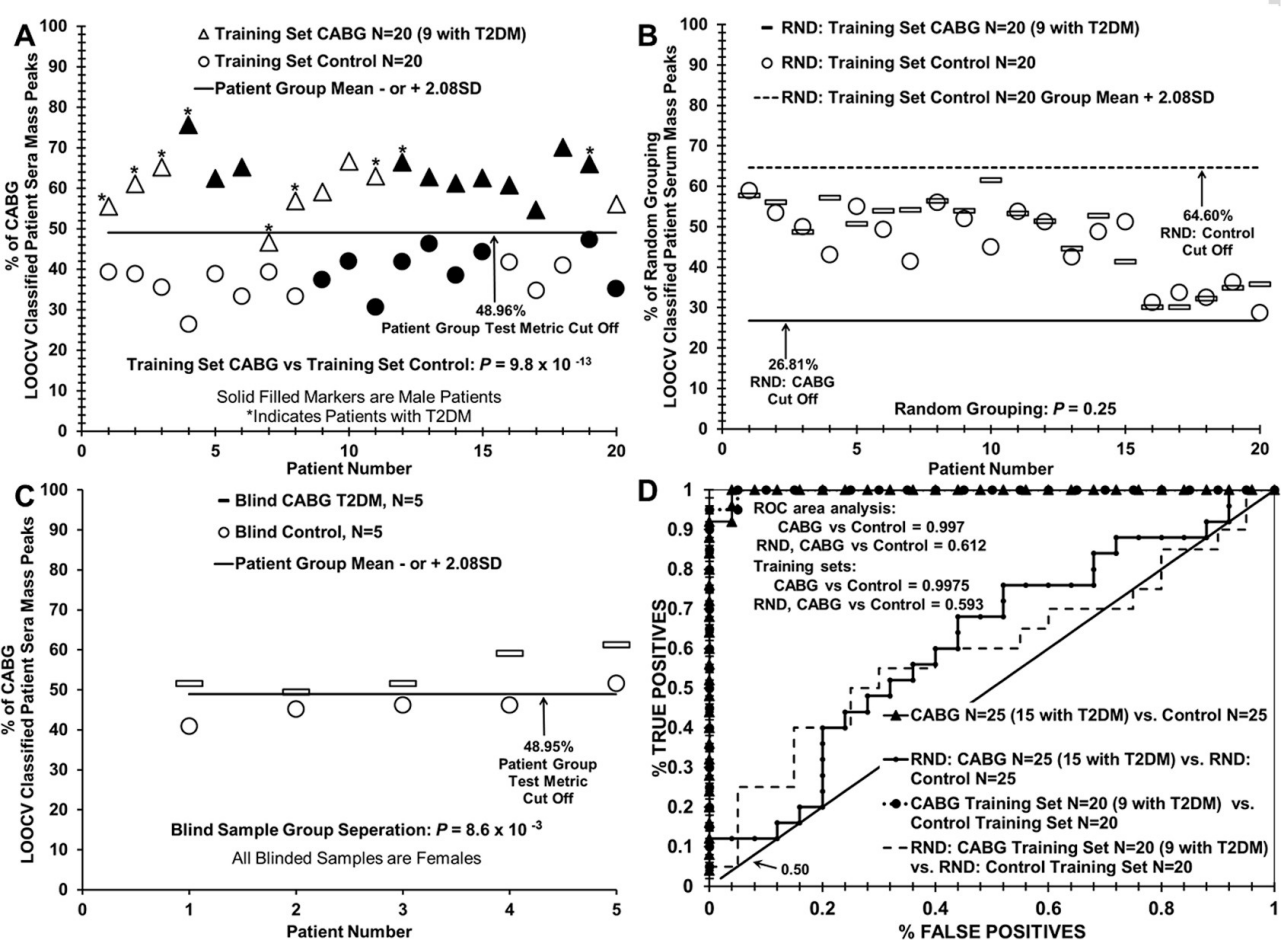
Analysis of “blind” or “left out” sets of subject sera samples using training a set composed of CABG patients versus control individuals. (A) Training set of serum discrimination of CABG patients (triangles) from controls (circles) by % of CABG LOOCV classified mass peaks; one false negative is observed. (B) Non-serum sample discrimination observed when the two different sample groups in panel A are mixed together randomly followed by the same LOOCV mass peak analysis. (C) Assessing the ability of the training set in panel A to correctly discriminate a blinded group of ten samples; 9 out of 10 samples were correctly identified. (D) Receiver operator characteristic (ROC) curve analysis of LOOCV data in Fig 4 and Fig 5. ROC analysis exhibits high sensitivity (true positive rate) of the group binary comparisons in Fig 4 panel A and Fig 5 panel A, and the near random results for the inter-group sample randomizations in Fig 4 panel B and Fig 5 panel B; the horizontal line exhibits a complete random result.

**Fig 6:**
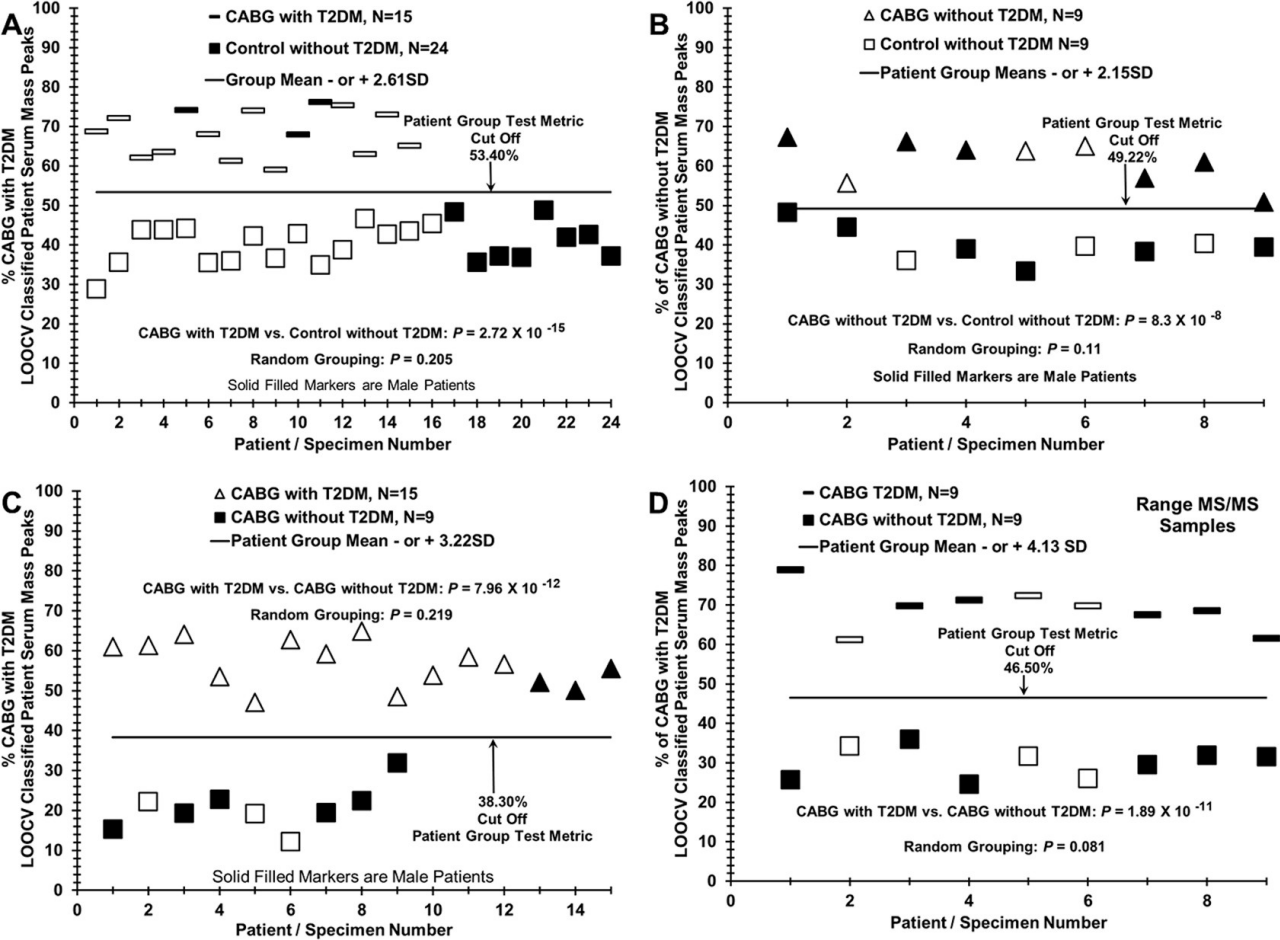
Distinguishing sera of CABG T2DM patients versus controls and CABG patients without T2DM. (A) Sera discrimination of patients with CABG with T2DM (dashes) versus sera from control individuals (squares) by % of LOOCV classified mass peak analysis. (B) Sera discrimination of patients with CABG without T2DM (triangles) versus sera from control individuals (squares). (C) Sera discrimination of CABG patients with T2DM (triangles) from CABG patients without T2DM (squares). (D) Discrimination of the sera samples in the 9 versus 9 grouping of patients with CABG plus T2DM (dashes) versus CABG without T2DM (squares) used in tandem MS/MS peptide/protein analysis exhibited in Table 3 and S1 Table.

Fig 3 panel B exhibits a number of significant ESI- MS mass peaks over a narrow mass range 800–925 m/Z used to discriminate sera from patients to undergo CABG surgery (solid line) from controls (dash line). The total MS range used in this study is 400–1908 m/Z. These significant (p <0.05) peak area means, as well as all the others in the total range used, differ between the CABG (N = 25) and control (N = 25) groups. Serum mass peak mean areas (higher value) from CABG patients include m/Z 814, 836, and 852. Peak area values higher for control individuals include 863, 874, and 911. This m/Z region is only one of many analysed (total range 400–1908 m/Z). The large number of significant peak differences over the larger range likely contribute to the disease discrimination ability of this technology [9–11]. Fig 3 panel B also exhibits the approach for categorizing/scoring significantly differing mass peaks as either CABG patients or controls using the PCV metric described in the Methods, Statistical Analysis. These data are used for construction of the “% of disease-specific LOOCV classified mass peaks” from the LOOCV peak assignments in the binary group serum discrimination studies (Figs 4–6). The LOOCV process helps mitigate a phenomenon termed “over-fitting” which can result from assigning relatively large amounts of experimental data to two groups, e.g. CABG or controls [18–20].

Fig 4 panel A illustrates the ability of this serum ESI-MS platform to distinguish sera from patients about to undergo the CABG procedure from control individuals without apparent CAD. When the “% of CABG LOOCV classified patient serum mass peaks” (obtained using the PCV analysis in Fig 3 panel B) is plotted versus patient number, a distribution plot is obtained in which a clear demarcation is observed between CABG patients (triangles) versus controls (circles). 129 to 140 LOOCV selected mass peaks were utilized for this group discrimination. The group separation has excellent 1.89 standard deviation (SD) variation from both the CABG and control group LOOCV % means, yielding a cut-off value of 46.98% CABG LOOCV classified mass peaks. The cut off is used for determining false positive and false negative test metric rates in Table 2.

Patient sample % scores above the cut off are considered to be identified as CABG samples and patient sample score below or equal the cut off are considered to be identified as control samples. The *p* value for this distribution difference is very low (10^−18^ range). This value becomes much higher moving toward non-significance (0.05) when these two subject groups are mixed together in random fashion and processed by the same LOOCV mass peak analysis. Plotting of this 0.05 *p* value discrimination is exhibited in Fig 4 panel B, demonstrating that no serum sample discrimination is observed at this non-significant *p* value (significant if *p* ≤ 0.05). This very large increase in *p* value upon randomization is consistent with minimal over-fitting, and supports the presence of a physiological basis for the original binary group discrimination. Fig 4 Panel B also identifies the majority of samples as members of both groups which is indicative of failure to assign correct grouping upon sample randomization (RAND). All samples below the dashed line are identified as samples of the RAND control group. All samples above the solid line are identified as samples of the RAND CABG group. The removal of known patient pathology after intermixing of samples fails to provide a reasonable group separation for the same number of peaks and cut off factors used in the construction of Fig 4 panel A.

### Use of ESI-MS to identify blinded CABG patient or control sera using a known training set

Although the present study is retrospective, prospective analyses are a future goal of this work. A blinded validation experiment testing the discriminatory power of the ESI-MS serum profiling platform in distinguishing sera of CABG patients or controls from each other is exhibited in Fig 5. This distinction is important as this methodology could be developed into a minimally invasive aid for monitoring CVD/CAD/T2DM development and progression. Toward this end, an initial validation test was conducted by removing 5 CABG with T2DM sera samples (dashes) and 5 control samples (circles) from each group of 25. The remaining samples were used as a “training set” of 20 each of the respective CABG (triangles) and control (circles) groups, and was subjected to LOOCV binary comparison Fig 5 panel A for significant peak selection. The *p* value for this training discrimination is still very low in the 10^−13^ range, and randomization of the samples raises the value toward insignificance 0.25, Fig 5 panel B. This training set binary discrimination Fig 5 panel C of the blinded samples does not display perfect sample discrimination as one control sample is above the cut-off value of 48.95%. Importantly, this blinded test discrimination Fig 5 panel C of the 10 “left out” samples, identified 9 out of the 10 to their correct patient groups, with one control sample identified as CABG. The *p* value for this test discrimination is a respectable, for a small sample size, 10^−3^ range. Fig 5 Panel D exhibits the ROC (receiver operator characteristic) curves for data in panels A and C, and the high true positive rate is exhibited for the training set and near randomness (curve closest to diagonal) for the randomized samples in panel B.

### Distinguishing sera of CABG patients with T2DM from control individuals and from CABG patients without T2DM

The ESI-MS methodology presented here could possibly be developed into a minimally invasive aid for monitoring CVD/CAD development and progression in the presence or absence of T2DM. Data in Fig 6 contributes to the evidence for the above statement when partnered with Fig 4 and Fig 5. Panel A, in both figures, depicts CABG patients with T2DM being distinguished from controls, and then a reduced number comparison for CABG patients without T2DM versus controls in panel B of both figures. Sample inter-group randomization followed by the identical LOOCV discrimination processes in both cases yield large reductions in discriminatory significant *p* values to random values above 0.05. This suggests physiological differences are contributing to the original binary group discriminations. Fig 5 panel C and Fig 5 panel D exhibit binary comparisons of CABG with T2DM versus CABG without T2DM at two different sample sizes, with the latter being a 9 versus 9 comparison. Again the randomization groupings are above the 0.05 non-significance value. The 9 versus 9 CABG with or without T2DM samples were used for the range MS/MS peptide/protein structure analyses (Table 3).

**Table 3:** 58 Protein/peptides identified by MS/MS in 4 or more sera from CABG patients with and without T2DM.

Symbol	T2DM: no T2DM	Symbol	T2DM: no T2DM	Symbol	T2DM: no T2DM
	# Sera (# hits)		# Sera (# hits)		# Sera (# hits)
IGH ^a^	9 (129) : 8 (601)	LRP1 ^a^^,^^b^^,^^c^^,^^d^	5 (60) : 4 (38)	CSMD2 ^d^	4 (56) : 1 (2)
IGL ^a^	9 (141) : 8 (140)	MUC17 ^d^	5 (57) : 4 (38)	PRRC2B	4 (56) : 1 (11)
IGK ^a^	8 (30) : 7 (162)	MUC2 ^a^^,^^b^	5 (55) : 4 (26)	MEGF6	0 (0) : 4 (41)
MTMR9 ^b^^,^^c^	8 (66) : 6 (142)	HERC2	1 (15) : 5 (48)	TENM3 ^a^	0 (0) : 4 (40)
TRB ^a^	7 (122) : 8 (132)	FCGBP ^d^	5 (47) : 4 (35)	THSD7B	4 (39) : 0 (0)
FBN3 ^b^^,^^d^	0 (0) : 7 (97)	ZAN	5 (36) : 2 (15)	FBXO34 ^d^	4 (36) : 0 (0)
HCFC2	7 (73) : 7 (83)	MACF1 ^a^^,^^b^^,^^d^	4 (230) : 3 (18)	MUC5B ^b^	4 (34) : 2 (12)
TRA ^a^^,^^d^	4 (27) : 7 (52)	LAMA2 ^d^	4 (132) : 2 (30)	KIF13B	0 (0) : 4 (32)
EBF4 ^a^	6 (238) : 0 (0)	NUP153 ^d^	3 (71) : 4 (120)	DNAAF2	4 (29) : 0 (0)
CORO7 ^c^	5 (107) : 6 (115)	DNAJC5	4 (99) : 0 (0)	OTOGL	4 (29) : 1 (5)
MUC19	6 (74) : 4 (23)	VPS13D ^a^^,^^c^	4 (98) : 0 (0)	IGHA2 ^a^^,^^d^	4 (26) : 1 (9)
MUC16 ^b^^,^^d^	6 (55) : 3 (32)	FBN1 ^d^	4 (93) : 2 (60)	LOC388282	0 (0) : 4 (24)
CORIN ^b^^,^^d^	5 (111) : 0 (0)	TNXB ^d^	3 (34) : 4 (90)	MALRD1 ^b^	1 (3) : 4 (24)
SYNE1 ^d^	5 (98) : 3 (65)	HSPG2 ^d^	4 (86) : 2 (33)	SCNN1B ^d^	4 (24) : 0 (0)
FBN2 ^b^^,^^d^	5 (93) : 0 (0)	MUC5AC ^a^^,^^b^	4 (74) : 3 (35)	NOTCH2 ^a^^,^^d^	4 (23) : 2 (21)
RELN ^d^	2 (17) : 5 (87)	CUBN ^d^	4 (68) : 0 (0)	CD5L ^a^^,^^b^^,^^c^^,^^d^	4 (22) : 1 (2)
VWF ^b^^,^^d^	0 (0) : 5 (73)	ANKRD17 ^d^	4 (65) : 0 (0)	CACNA2D2 ^d^	4 (20) : 0 (0)
TTN ^d^	5 (71) : 2 (38)	SCUBE1 ^d^	4 (65) : 3 (37)	INHBB ^b^^,^^c^^,^^d^	1 (3) : 4 (17)
SPG11	1 (38) : 5 (70)	DPYD	0 (0) : 4 (62)		
SSPO	5 (25) : 4 (67)	ERVH48-1	4 (60) : 2 (24)		

^a^ (immune / inflammation)

^b^ (T2DM / metabolic syndrome, insulin resistance)

^c^ (obesity)

^d^ (CVD / CAD / hypertension / atherosclerosis)

### Test metric data for CABG versus controls versus CABG with T2DM serum profiling comparisons

Table 2 summarizes the test metrics for the binary group discriminatory and randomized LOOCV data exhibited in Figs 4–6. The pathological groups tested in binary fashion from these Figs are listed in the far-left column. These metrics include the % LOOCV classified mass peak means and standard deviations (SD) for all the group comparisons (left column), with respect to the specific Figs and panels (far right column). Nomenclature from predictive value theory is presented, e.g., test sensitivity, specificity, etc., as well as true pathology and random grouping *p* values [23, 24]. The “% LOOCV MS peaks” means and their standard deviation (SD) are all well separated and have narrow SD boundaries for all the groups tested. Test sensitivities (true positive rate) and specificities (true negative rate) range from 1.0 to 0.80 respectively. The potential presence of physiological processes accounting for the original binary group distribution differences are indicated by the very large increases in *p* values when the groups are randomized. In addition, a Cohen’s *d* effect size value is provided in Table 2. “Effect size” refers to the mean differences for the binary group LOOCV discriminations taking into consideration the SD values and percent group mean differences [18, 23].

This Cohen’s *d* value is an indirect measure of statistical power (ability to detect type II errors-false negatives) of the sample sizes employed in a study. The large Cohen’s *d* values exhibited here bolster the reliability and power (estimated from these effect sizes (24) to be > 0.90) of the sample sizes exhibited in Table 2.

### Phenotype assessment of CAD/CABG patients with and without T2DM using MS/MS serum peptide/protein identifications and bio-informatic cell/biochemical pathway analysis

It is important to identify biomolecules in the peripheral blood that change with different disease states like CAD and CAD plus T2DM as this information could be useful in monitoring these disease states and in providing phenotypic, mechanistic, and therapeutic insights into those diseases. Table 3 exhibits the top 58 peptides/proteins identified by tandem MS/MS of sera (9 samples each, 6 male, 3 female patients) for the Fig 6D CABG patient with T2DM versus CABG patient without T2DM binary comparison. A serum mass peak range of 900–1008 m/Z, in unit Dalton values was examined for this analysis because previous experience indicated this was a productive ionisable region of the mass spectrum. The peptides/proteins are listed by their corresponding protein name/abbreviation, and are ranked by their serum presence (out of 9 samples) and numbers of MS/MS “hits” (individual peptide identifications, a semi-quantitative measure). Exhibited here are all the peptides/proteins identified in 4 or more out of 9 serum samples. A total of 139 different peptide/proteins were identified in 3 or more out of 9 samples (S1 Table). Because of keratin contamination from needle puncture through skin/hair layers during the blood draws, keratin peptides/proteins are not listed in these tables.

**Fig 7:**
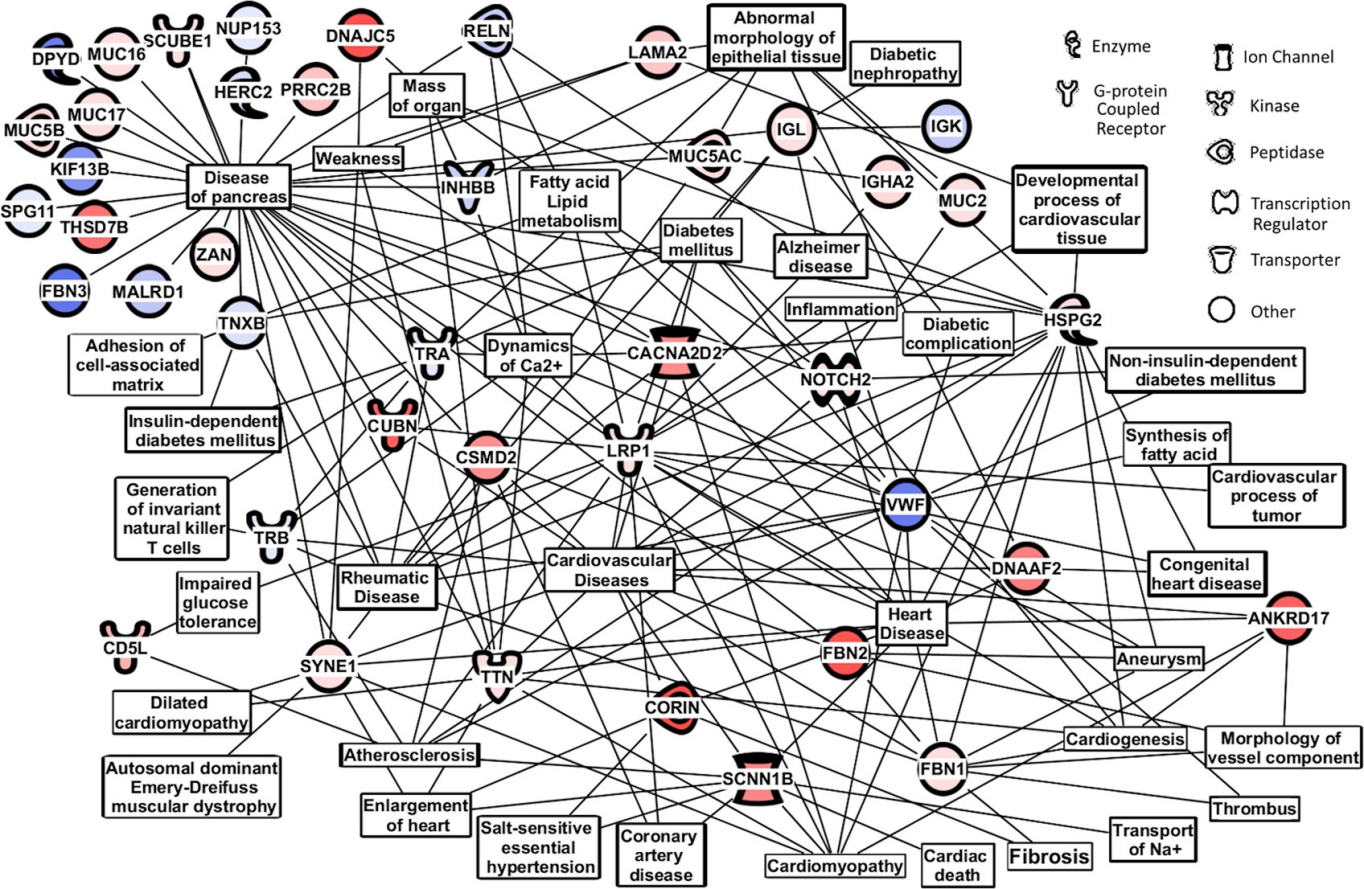
Physiological/cellular pathways and serum peptides/proteins found changing in Table 3 comparing CABG patients with and without T2DM. Affected/altered physiological/cellular pathways using the 58 serum peptide/protein assignments from Table 3 that distinguished CABG with T2DM patients from CABG patients without T2DM from the sera samples used in Fig 6 panel D. Analysis was performed using Ingenuity Pathway Analysis (IPA) bioinformatics software (Qiagen, Inc.).

A PubMed/Medline literature examination of the peptides/proteins and their functions listed in Table 3 reveals overall phenotypes evident from the CABG versus CABG+T2DM sera comparison as follows: cardiovascular disease (50%), immune/inflammation (26%), T2DM (24%), and obesity (12%), on a per individual serum sample basis. Sixteen percent of the serum samples in Table 3 fall into the CVD+T2DM joint phenotype. Notable peptides/proteins in this group, as evidenced by previous literature reports, include FBN3, MUC16, CORIN, FBN2, VWF, LRP1, MACF1, CD5L, INHBB. CORIN (Atrial natriuretic peptide-converting enzyme) and Mucin 16 (MUC16), which are both observed elevated in both sera numbers and MS/MS “hits” in the CABG+T2DM category in Table 3, were previously suggested to be potential biomarkers for monitoring heart disease and heart failure [26, 27]. CORIN is also proposed to be a possbile biomarker for cardiovascular disease complications in T2DM patients [28]. It is very important to monitor T2DM patients for cardiovascular disorders, which is an eventual goal of the present study. CORIN also likely has roles in fibrosis in cardiomyopathy and atherosclerosis [29]. Two peptide/proteins in Table 3 found in all four phenotype categories (CVD, T2DM, inflammation, obesity) are LRP1 (low-density lipoprotein receptor related protein 1) and CD5L (CD5 Molecule Like). Both are elevated in the CABG T2DM sera in this Table. LRP1 has a multitude of direct roles in lipid and cholesterol metabolism, glucose homeostasis, inflammation, and atherosclerosis [30, 31]. CD5L is involved in lipid homeostasis (especially with respect to obesity and inflammatory responses), atherosclerosis, insulin resistance, and metabolic syndrome [32]. CORO7 (Coronin 7) is another peptide/protein listed in Table 3 that has previously observed roles in obesity and body weight regulation [33]. These and other identified peptides and represented proteins in Table 3 could potentially be discriminatory biomarkers for these disorders as well as possible therapeutic targets for CAD and T2DM and suggest their future study could be of value. It is noted that the data in Table 3 are the result of a RANGE MS/MS analysis Fig 7 of the same m/Z unit Dalton mass peaks (900 to 1008) in both the CABG and CABG+T2DM patient sera samples. This analysis gives a potential overview of what is present at the peptide level comparing sera from these patients and their respective disease states.

Fig 7 exhibits cellular/biochemical pathways that the bio-informatic software package Ingenuity Pathway Analysis (IPA) indicated are affected/changing from the list of the 58 proteins differentially present in the CABG vs CABG+T2DM comparison in Table 3. Inputs are also present from the Medline/PubMed analysis described in Table 3. Major pathways affected (by numbers of pathway connections) include those associated with Pancreas Disease, Heart Disease, Cardiovascular Disease, Rheumatic Disease, Fatty Acid Lipid Metabolism, Atherosclerosis, Calcium Dynamics, Cardiomyopathy, Alzheimer’s Disease, Inflammation. The Alzheimer’s disease connection appearing in this CABG vs CABG+T2DM comparison is interesting as Alzheimer links to both T2DM and atherosclerosis have been previously reported [34, 35]. Major protein hubs in Fig 7 include LRP1, VWF (von Willibrand factor), HSPG2 (heparan sulfate proteoglycan 2), SCNN1B (sodium channel epithelial 1 beta subunit), NOTCH2, CORIN. Fig 7 is expanded in S1 Fig by inclusion of twice as many peptides/proteins, including a number of 3 or better sera out of 9, found in S1 Table. Many of the pathway and protein hubs and connections from Fig 7 are maintained and expanded, but also additional important ones like apoptosis and morbidity/mortality. A separate morbidity and mortality IPA figure is in S2 Fig exhibiting the large number of proteins present with that phenotype.

## Discussion

Coronary heart disease (CHD), of which coronary artery disease (CAD) is the major component, is the largest cause of death in developed countries, and is becoming the leading cause of mortality and morbidity in developing countries [36]. CAD is a major factor for the long-term health prognosis of patients with DM, and is associated with a 2 to 4-fold increase in mortality risk of DM patients [37]. CAD is the main cause of death in both type 1 and type 2 DM [38]. About 70% of people 65 years of age and older with DM will die from some form of heart disease [39]. Monitoring CAD usually involves invasive and expensive procedures like angiograms. Stress tests are less invasive but less informative as well, in most cases leading to angiograms. It is important to obtain minimally invasive and less costly but accurate measures to help monitor CAD in high-risk individuals, especially those with type 1 or type 2 DM. Peripheral blood biomarkers and related procedures are one approach to less costly identification and monitoring of CAD associated with T2DM [6]. A more recent study emphasizing CAD monitoring specifically in T2DM patients versus T2DM patients without CAD, using a variety of commercially available antibody arrays, was able to demonstrate that multiple biomarker classifiers were involved, principally in inflammation, insulin resistance, endothelial cell dysfunction, and lipolysis and fatty acid pathways in progression of CAD in T2DM patients [40].

The development of such diagnostic approaches and monitoring aids would allow improved screening of these conditions, helping with patient treatment and prognosis. Also such analyses can assist in understanding the underlying biochemistry, progression, and complicating factors in these disorders. In the present study we demonstrate the ability to distinguish serum samples (at the N values listed) from control individuals, CABG, and CABG plus T2DM patient groups. The approach employed utilizes an all-liquid unfractionated serum mass profiling procedure to analyse serum mass peaks and biomolecules. This technology was previously successful in examining and distinguishing early stages of various cancers and neurological disorders [7–10]. This methodology is straightforward, involving serum isolation from peripheral blood, dilution and injection into an electrospray ionization mass spectrometer, followed by software mass peak analysis. The serum is not fractionated nor excessively handled, making this methodology potentially a simple serum biomarker platform available for CAD and CAD+T2DM discrimination. This minimal handling leads to less chance for the introduction of artifacts, and ease of use by clinical staff.

The hypothesis guiding this work is that serum mass peaks, resulting from tissues shedding/secreting biomolecules into the peripheral blood, will reflect specific physiological changes associated with different disease states such as CAD or CAD+T2DM. These diseases can possibly be monitored in steady state using this methodology because their physiological differences are hypothesized to have caused measurable biomolecule changes in the peripheral blood due to host systemic responses, homeostasis and defense responses, stress mechanisms, and direct inputs from diseased tissues. The large number of different identifiers (mass peaks) used by this methodology, differing from a number of other biomarker platforms in this regard, is likely helping its specific disease monitoring ability [9–11]. To help reduce/ameliorate potential over-fitting of large serum mass peak data sets produced in this study, leave one [serum sample] out cross validation (LOOCV) was utilized as described in Fig 4B and in the Methods. To further check for over-fitting and a physiological basis for true-pathology binary group comparisons and discriminations, serum samples between binary groups were randomized followed by the same LOOCV mass peak analysis. This procedure resulted in moving the very low *p-*values for true-pathology binary group discriminations Fig 4 panel A, Fig 5 panel A, Fig 6 panel A, and Fig 6 panel C toward or above non-significant values which upon examination have no group discrimination value Fig 4 panel B and Fig 5 panel B. Such randomization results are consistent with disease-specific inputs into the peripheral blood having a role in the specific group discriminations [9, 10].

With the success for CAD vs CAD+T2DM patient and other group discriminations described in this study, larger sample sizes in the future will be assessed as they become available. However, in the present study the large “effect sizes” for these binary group comparisons (differences in mean mass peak areas and standard deviations for the two different groups in question (Table 2, measured in part by Cohen *d* values) yield statistical powers in the above 0.95 ranges). This lends credibility to the discriminations even at reduced sample sizes, and establishes their validity and portends well for future studies with larger sample sizes.

Most of the LOOCV mass peak area values being analysed in this study, e.g., from around 500 to 1200 m/Z, likely encompass the lower mass peptide “serome” resulting from differential host tissue/organ exoprotease activities and other cell/tissue signaling activities [12, 13]. The “serome” is composed of a large number of different biomolecules. The specific disease monitoring and discriminating ability of this ESI-MS platform is likely due to the large number of distinguishing components analysed at the same time. It is known that the larger numbers of different components analysed in a biomarker analytical platform, the greater the disease discriminatory powers of the platform [9–11]. Since fairly evident biomolecule changes are being observed from apparent small physiological inputs, one would need to hypothesize a mechanism(s) to account for such apparent amplification of small signal(s) from small starting inputs. Possible mechanisms here could involve “alarmin”-like molecules believed to be shed/secreted by cells which have been damaged/altered in some fashion, which in turn bind to signal transduction pathway receptors to activate in a synergistic and cooperative manner more extensive innate defense/stress responses [13]. This biomolecule complexity in the “serome” brings up the additional question of what might be the physiological basis and associated changes for the sera group discriminations being observed in this study. In order to aid in the identification of such physiological differences which possibly contribute to the patient pathology and serum discriminations, this ESI-MS platform also has the ability to “target”, by tandem MS/MS structure determinations, similar and different mass peaks in both CAD and CAD+T2DM patient sera to observe how biomolecules are varying between the two disease states and give clues about their respective pathologies. This represents another unique aspect of this ESI-MS methodology: disease identification and monitoring as well as disease understanding through identification of a wide variety of biomolecules involved in disease mechanisms. With the platform described here, this can be performed on a single instrument with a very small sample of a bodily fluid. The identification of such biomolecules and biochemical pathways can aid in further novel therapeutic and biomarker development. A survey of serum mass peak tandem MS/MS analyses, over a defined mass peak range (900–1008 m/Z), are presented in Table 3 for the top 58 peptides/proteins respectively as judged by sera presence (out of 9 patients/samples) in CAD and/or CAD+T2DM and number of MS/MS ‘hits”/peptide identifications.

Peptide fragments of immunoglobulin proteins with their high prevalence in sera are at the top of the Table 3 list with respect to sera presence and MS/MS hits. Of interest, prominent CVD/CAD or T2DM phenotypes are evident in Table 3 with 74% of the 58 peptides/proteins listed having these functions as determined from published literature searchs of PubMed/Medline. Sixteen percent of the peptides/proteins in this Table are associated with both a CAD/CVD and T2DM joint phenotype. Several peptides/proteins in this list have direct applicability to previous studies on CAD and T2DM and were mostly detectable in sera from patients with CAD plus T2DM (e.g., CORIN, MACF1, CD5L). CORIN (Atrial natriuretic peptide-converting enzyme) is found in 5 out of 9 CAD+T2DM patient sera samples in Table 3 and in none of the CABG patients without T2DM. CORIN is a transmembrane protease that proteolyzes cardiac natriuretic peptides. CORIN is shed from the cell surface into the peripheral blood [26]. This protein was previously proposed to be a biomarker for complications in CAD+T2DM patients [28]. Interestingly, the protein has roles in cardiomyopathy and atherosclerosis possibly through fibrosis[29]. This process of fibrosis and scarring is precisley what we have observed in the endothelial vasculature and smooth muscle in the affected coronary artieries in the CAD plus T2DM patients in the present study Fig 2, panel I. MACF1 (Microtubule actin cross-linking factor 1) appears to be an important pleiotropic factor involved in and at the cross-roads of metabolic syndrome, inflammation, T2DM, and CVD [41]. This protein also appears to have a role in cardiac structural changes associated with cardiomyopathy[42]. CD5L (CD [cluster of differentiation] 5 molecule like) is an important soluble protein proposed to interconnect inflammation with obesity, lipid metabolism and lipidome, insulin resistance, and atherosclerosis [32]. Lipid dysfunction and deposition is noted in our CABG+T2DM patients with their much enlarged abdominal fat cells Fig 1, panel I.

The peptides/proteins listed in Table 3, and their differences between the CABG and CABG+T2DM patient sera, were inserted into Ingenuity Pathway Analysis (IPA) bioinformatics software to possibly identify what known biological pathways appear to be influenced by these peptides/proteins and their changes in these two disease states Fig 7. Of interest epilepsy/seizure effects are observed in cell pathway effects observed by IPA analysis of the top 58 peptides/proteins identified by MS/MS Table 3 which were found in 4 or more sera out of 9 samples per patient. This IPA analysis highlights the importance of cardiovascular and coronary artery disease, atherosclerosis, pancreatic disese, heart disease, DM, fatty acid and lipid metabolism, Alzheimer’s disese, inflammation, calcium dynamics, and fibrosis cell/biochemical pathway effects taking place in the CABG vesus CABG plus T2DM serum comparisons. Many of these affected pathways are retained in IPA S1 Fig which uses the top 116 peptides/proteins found in 3 or more out of 9 different patient sera exhibited in the S1 Table. Of interest, apoptosis becomes much more apparent in this figure which is in line with the loss of cellularity observed in the coronary vascular endothelium and smooth muscle in the aortic punches used for the vein bypass procedure graph, principally for the CABG+T2DM patients Fig 2, part I. Also cell pathways associated with morbidity and mortality are much more affected using the larger peptide/protein database S1 Fig and S2 Fig. This indicates the seriousness of the health conditions of these patients with CAD and the need for the CABG procedure. Observing the presence of these known CVD/CAD and T2DM-related disease phenotypes in Fig 7, S1 Fig and S2 Fig lends credence to the ability of this serum mass profiling methodology and platform described here to help distinguish these groups Fig 4, Fig 5, Fig 6 and decipher pathologies, which is consistent with the serum mass profiling hypothesis guiding these studies. By providing evidence of fibrosis, apoptosis, calcium/sodium ion mechanisms, and dementia in CAD, this study provides basic observations which could open up new avenues of thought and future possible research concerning CAD and associated T2DM. Future studies will examine larger numbers of serum samples in these contextes, and also test for peptide/protein presence, e.g., using identifications in Table 3 and S1 Table, in CABG and CABG with T2DM patients sera using immunoassays.

## Supporting information

S1 Table 139 Protein/peptides identified by MS/MS in 3 or more sera from CABG patients with and without T2DM. (DOCX)

S1 Fig Physiological/cellular pathways and serum peptides/proteins found altered in CABG patients with and without T2DM from the top 116 peptides/proteins found in S1 Table. (TIF)

S2 Fig IPA analysis using peptide/protein comparative data in S1 Table of CABG patients with and without T2DM, with emphasis on morbidity/mortality. (TIF)

S1 File Data Peaks used in each figure. (XLSX)

S2 File Data Patients used in each figure. (XLSX)

S3 File Data Patient health characteristics. (XLSX)

S4 File Data MSMS results used in tables. (XLSX)

S5 File Data initial raw exported injection intensity. (XLSX)

S6 File Data normalized signal intensity. (XLSX)

S7 File Data Peaked injection areas. (XLSX)

S8 File Data peaked normalized injection areas. (XLSX)

S9 File Data averaged peak areas. (XLSX)

S1 Raw data Patient triplicate MS ThermoFisher. (ZIP)

S2 Raw data Patient triplicate MS ThermoFisher. (ZIP)

S3 Raw data Patient triplicate MS ThermoFisher. (ZIP)

S4 Raw data Patient triplicate MS ThermoFisher. (ZIP)

S5 Raw data Patient triplicate MS ThermoFisher. (ZIP)

S6 Raw data Patient triplicate MS ThermoFisher. (ZIP)

S7 Raw data Patient triplicate MS ThermoFisher. (ZIP)

S8 Raw data Patient triplicate MS ThermoFisher. (ZIP)

S9 Raw data Patient triplicate MS ThermoFisher. (ZIP)

S10 Raw data Patient triplicate MS ThermoFisher. (ZIP)

S11 Raw data Patient triplicate MS ThermoFisher. (ZIP)

S12 Raw data Patient triplicate MS ThermoFisher. (ZIP)

S13 Raw data Patient triplicate MS ThermoFisher. (ZIP)

S14 Raw data Patient triplicate MS ThermoFisher. (ZIP)

S15 Raw data Patient triplicate MS ThermoFisher. (ZIP)

S16 Raw data Patient triplicate MS ThermoFisher. (ZIP)

S17 Raw data Patient triplicate MS ThermoFisher. (ZIP)

S18 Raw data Patient triplicate MS ThermoFisher. (ZIP)

S19 Raw data Patient triplicate MS ThermoFisher. (ZIP)

S20 Raw data Patient triplicate MS ThermoFisher. (ZIP)

S21 Raw data Patient triplicate MS ThermoFisher. (ZIP)

S22 Raw data Patient triplicate MS ThermoFisher. (ZIP)

S23 Raw data Patient triplicate MS ThermoFisher. (ZIP)

S24 Raw data Patient triplicate MS ThermoFisher. (ZIP)

S25 Raw data Patient triplicate MS ThermoFisher. (ZIP)

S26 Raw data Patient triplicate MS ThermoFisher. (ZIP)

S27 Raw data Patient triplicate MS ThermoFisher. (ZIP)

S28 Raw data Patient triplicate MS ThermoFisher. (ZIP)

S29 Raw data Patient triplicate MS ThermoFisher. (ZIP)

S30 Raw data Patient triplicate MS ThermoFisher. (ZIP)

S31 Raw data Patient triplicate MS ThermoFisher. (ZIP)

S32 Raw data Patient triplicate MS ThermoFisher. (ZIP)

S33 Raw data Patient triplicate MS ThermoFisher. (ZIP)

S34 Raw data Patient triplicate MS ThermoFisher. (ZIP)

S35 Raw data Patient triplicate MS ThermoFisher. (ZIP)

S36 Raw data Patient triplicate MS ThermoFisher. (ZIP)

S37 Raw data Patient triplicate MS ThermoFisher. (ZIP)

S38 Raw data Patient triplicate MS ThermoFisher. (ZIP)

S39 Raw data Patient triplicate MS ThermoFisher. (ZIP)

S40 Raw data Patient triplicate MS ThermoFisher. (ZIP)

S41 Raw data Patient triplicate MS ThermoFisher. (ZIP)

S42 Raw data Patient triplicate MS ThermoFisher. (ZIP)

S43 Raw data Patient triplicate MS ThermoFisher. (ZIP)

S44 Raw data Patient triplicate MS ThermoFisher. (ZIP)

S45 Raw data Patient triplicate MS ThermoFisher. (ZIP)

S46 Raw data Patient triplicate MS ThermoFisher. (ZIP)

S47 Raw data Patient triplicate MS ThermoFisher. (ZIP)

S48 Raw data Patient triplicate MS ThermoFisher. (ZIP)

S49 Raw data Patient triplicate MS ThermoFisher. (ZIP)

S50 Raw data Patient triplicate MS ThermoFisher. (ZIP)

S51 Raw data Patient MSMS ThermoFisher. (ZIP)

S52 Raw data Patient MSMS ThermoFisher. (ZIP)

S53 Raw data Patient MSMS ThermoFisher. (ZIP)
