# Supplementary material for: Serum discrimination and phenotype assessment of coronary artery disease patents with and without type 2 diabetes prior to coronary artery bypass graft surgery
Source: PLoS One. 2020 Aug 5;15(8):e0234539. doi: 10.1371/journal.pone.0234539 (PMC7527241; doi:10.1371/journal.pone.0234539)
Supplement: S1 Table — (DOCX) [file pone.0234539.s001.docx]

**S1 Table. 139 Protein/peptides identified by MS/MS in 3 or more sera from CABG patients with and without T2DM**

| **Symbol** | **T2DM : no T2DM** | **Symbol** | **T2DM : no T2DM** | **Symbol** | **T2DM : no T2DM** |
| --- | --- | --- | --- | --- | --- |
|  | **# Sera (# hits)** |  | **# Sera (# hits)** |  | **# Sera (# hits)** |
| IGH | 9 ( 129 ) : 8 ( 601 ) | KIF13B | 0 ( 0 ) : 4 ( 32 ) | SVEP1 | 3 ( 39 ) : 2 ( 8 ) |
| IGL | 9 ( 141 ) : 8 ( 140 ) | OTOGL | 4 ( 29 ) : 1 ( 5 ) | ZFYVE16 | 3 ( 39 ) : 0 ( 0 ) |
| IGK | 8 ( 30 ) : 7 ( 162 ) | DNAAF2 | 4 ( 29 ) : 0 ( 0 ) | C11orf16 | 3 ( 38 ) : 0 ( 0 ) |
| MTMR9 | 8 ( 66 ) : 6 ( 142 ) | IGHA2 | 4 ( 26 ) : 1 ( 9 ) | TRPM7 | 0 ( 0 ) : 3 ( 38 ) |
| TRB | 7 ( 122 ) : 8 ( 132 ) | MALRD1 | 1 ( 3 ) : 4 ( 24 ) | NR3C2 | 3 ( 37 ) : 0 ( 0 ) |
| FBN3 | 0 ( 0 ) : 7 ( 97 ) | LOC388282 | 0 ( 0 ) : 4 ( 24 ) | SCRIB | 0 ( 0 ) : 3 ( 37 ) |
| HCFC2 | 7 ( 73 ) : 7 ( 83 ) | SCNN1B | 4 ( 24 ) : 0 ( 0 ) | LAMC1 | 3 ( 35 ) : 1 ( 5 ) |
| TRA | 4 ( 27 ) : 7 ( 52 ) | NOTCH2 | 4 ( 23 ) : 2 ( 21 ) | CEP192 | 3 ( 35 ) : 0 ( 0 ) |
| EBF4 | 6 ( 238 ) : 0 ( 0 ) | CD5L | 4 ( 22 ) : 1 ( 2 ) | COL4A3 | 3 ( 34 ) : 1 ( 22 ) |
| CORO7 | 5 ( 107 ) : 6 ( 115 ) | CACNA2D2 | 4 ( 20 ) : 0 ( 0 ) | PKD1 | 3 ( 33 ) : 1 ( 8 ) |
| MUC19 | 6 ( 74 ) : 4 ( 23 ) | INHBB | 1 ( 3 ) : 4 ( 17 ) | AGRN | 3 ( 33 ) : 0 ( 0 ) |
| MUC16 | 6 ( 55 ) : 3 ( 32 ) | TENM2 | 3 ( 101 ) : 1 ( 11 ) | ARID1A | 3 ( 32 ) : 2 ( 11 ) |
| CORIN | 5 ( 111 ) : 0 ( 0 ) | CD40LG | 3 ( 91 ) : 1 ( 4 ) | FCRL4 | 3 ( 32 ) : 0 ( 0 ) |
| SYNE1 | 5 ( 98 ) : 3 ( 65 ) | SORBS1 | 3 ( 87 ) : 1 ( 3 ) | SERINC2 | 3 ( 32 ) : 0 ( 0 ) |
| FBN2 | 5 ( 93 ) : 0 ( 0 ) | LRIG1 | 3 ( 84 ) : 0 ( 0 ) | SLC12A5 | 2 ( 21 ) : 3 ( 31 ) |
| RELN | 2 ( 17 ) : 5 ( 87 ) | MAMLD1 | 3 ( 84 ) : 0 ( 0 ) | MUC4 | 0 ( 0 ) : 3 ( 31 ) |
| VWF | 0 ( 0 ) : 5 ( 73 ) | NAV2 | 3 ( 75 ) : 0 ( 0 ) | NEK11 | 0 ( 0 ) : 3 ( 31 ) |
| TTN | 5 ( 71 ) : 2 ( 38 ) | SMG1 | 1 ( 13 ) : 3 ( 74 ) | APC | 3 ( 30 ) : 1 ( 9 ) |
| SPG11 | 1 ( 38 ) : 5 ( 70 ) | BAG6 | 3 ( 72 ) : 1 ( 5 ) | LRIG3 | 3 ( 30 ) : 1 ( 9 ) |
| SSPO | 5 ( 25 ) : 4 ( 67 ) | CSMD3 | 3 ( 66 ) : 3 ( 58 ) | VCAN | 1 ( 4 ) : 3 ( 30 ) |
| LRP1 | 5 ( 60 ) : 4 ( 38 ) | KIAA1324 | 3 ( 63 ) : 0 ( 0 ) | TBL3 | 3 ( 30 ) : 0 ( 0 ) |
| MUC17 | 5 ( 57 ) : 4 ( 38 ) | RASA2 | 3 ( 60 ) : 0 ( 0 ) | MEGF10 | 3 ( 28 ) : 1 ( 5 ) |
| MUC2 | 5 ( 55 ) : 4 ( 26 ) | NR4A1 | 0 ( 0 ) : 3 ( 58 ) | EMILIN1 | 3 ( 28 ) : 0 ( 0 ) |
| HERC2 | 1 ( 15 ) : 5 ( 48 ) | DST | 3 ( 57 ) : 2 ( 26 ) | MEGF11 | 3 ( 27 ) : 2 ( 18 ) |
| FCGBP | 5 ( 47 ) : 4 ( 35 ) | SCUBE2 | 3 ( 55 ) : 0 ( 0 ) | SLIT1 | 3 ( 27 ) : 1 ( 9 ) |
| ZAN | 5 ( 36 ) : 2 ( 15 ) | PCSK6 | 2 ( 20 ) : 3 ( 54 ) | DOCK11 | 3 ( 27 ) : 1 ( 7 ) |
| MACF1 | 4 ( 230 ) : 3 ( 18 ) | FRYL | 3 ( 54 ) : 1 ( 3 ) | DCLRE1C | 3 ( 27 ) : 0 ( 0 ) |
| LAMA2 | 4 ( 132 ) : 2 ( 30 ) | TECTA | 3 ( 19 ) : 2 ( 52 ) | NFX1 | 1 ( 17 ) : 3 ( 26 ) |
| NUP153 | 3 ( 71 ) : 4 ( 120 ) | CAND1 | 3 ( 52 ) : 0 ( 0 ) | FRAS1 | 3 ( 26 ) : 1 ( 5 ) |
| DNAJC5 | 4 ( 99 ) : 0 ( 0 ) | DAZAP1 | 3 ( 52 ) : 0 ( 0 ) | PARN | 0 ( 0 ) : 3 ( 26 ) |
| VPS13D | 4 ( 98 ) : 0 ( 0 ) | LMO7 | 3 ( 52 ) : 0 ( 0 ) | UXS1 | 2 ( 10 ) : 3 ( 25 ) |
| FBN1 | 4 ( 93 ) : 2 ( 60 ) | SHANK2 | 0 ( 0 ) : 3 ( 52 ) | TEP1 | 3 ( 25 ) : 1 ( 7 ) |
| TNXB | 3 ( 34 ) : 4 ( 90 ) | PAX5 | 1 ( 3 ) : 3 ( 49 ) | ITGB2 | 3 ( 25 ) : 0 ( 0 ) |
| HSPG2 | 4 ( 86 ) : 2 ( 33 ) | NFATC1 | 3 ( 49 ) : 0 ( 0 ) | ZFHX2 | 3 ( 25 ) : 0 ( 0 ) |
| MUC5AC | 4 ( 74 ) : 3 ( 35 ) | SLC4A5 | 0 ( 0 ) : 3 ( 47 ) | KMT2D | 3 ( 24 ) : 2 ( 14 ) |
| CUBN | 4 ( 68 ) : 0 ( 0 ) | LOC107985428 | 3 ( 46 ) : 1 ( 8 ) | BTBD7 | 0 ( 0 ) : 3 ( 24 ) |
| SCUBE1 | 4 ( 65 ) : 3 ( 37 ) | COL6A3 | 0 ( 0 ) : 3 ( 46 ) | DNAJC5G | 3 ( 24 ) : 0 ( 0 ) |
| ANKRD17 | 4 ( 65 ) : 0 ( 0 ) | NF1 | 0 ( 0 ) : 3 ( 45 ) | NCAPD2 | 0 ( 0 ) : 3 ( 24 ) |
| DPYD | 0 ( 0 ) : 4 ( 62 ) | STAG2 | 3 ( 45 ) : 0 ( 0 ) | LRP1B | 3 ( 23 ) : 2 ( 22 ) |
| ERVH48-1 | 4 ( 60 ) : 2 ( 24 ) | MUC21 | 3 ( 44 ) : 0 ( 0 ) | NOTCH3 | 3 ( 20 ) : 3 ( 23 ) |
| PRRC2B | 4 ( 56 ) : 1 ( 11 ) | STAB1 | 2 ( 43 ) : 3 ( 33 ) | MAN2C1 | 2 ( 10 ) : 3 ( 23 ) |
| CSMD2 | 4 ( 56 ) : 1 ( 2 ) | LTBP2 | 3 ( 31 ) : 3 ( 42 ) | HUWE1 | 3 ( 23 ) : 1 ( 9 ) |
| MEGF6 | 0 ( 0 ) : 4 ( 41 ) | TRPS1 | 3 ( 41 ) : 1 ( 7 ) | CDHR2 | 3 ( 23 ) : 0 ( 0 ) |
| TENM3 | 0 ( 0 ) : 4 ( 40 ) | LAYN | 3 ( 41 ) : 0 ( 0 ) | MUC3A | 3 ( 22 ) : 2 ( 6 ) |
| THSD7B | 4 ( 39 ) : 0 ( 0 ) | ZMIZ1 | 3 ( 40 ) : 0 ( 0 ) | FAT1 | 3 ( 22 ) : 1 ( 3 ) |
| FBXO34 | 4 ( 36 ) : 0 ( 0 ) | KMT2A | 1 ( 15 ) : 3 ( 39 ) | OPALIN | 0 ( 0 ) : 3 ( 22 ) |
| MUC5B | 4 ( 34 ) : 2 ( 12 ) |  |  |  |  |
|  | | | | | |
