# Supplementary figures and images for: Serum discrimination and phenotype assessment of coronary artery disease patents with and without type 2 diabetes prior to coronary artery bypass graft surgery
Source: PLoS One. 2020 Aug 5;15(8):e0234539. doi: 10.1371/journal.pone.0234539 (PMC7527241; doi:10.1371/journal.pone.0234539)

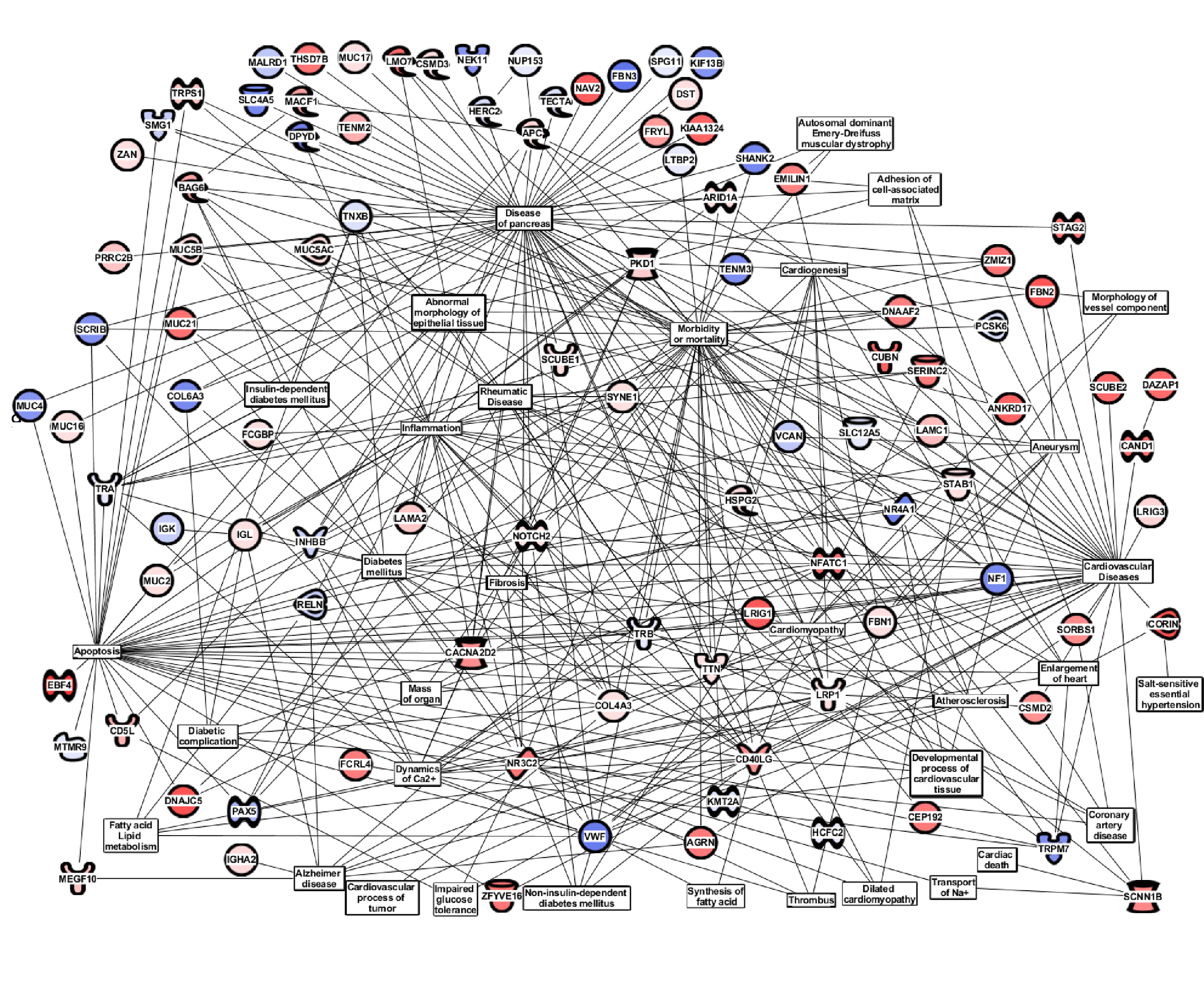

Supplement: S1 Fig — (TIF) [file pone.0234539.s002.tif]

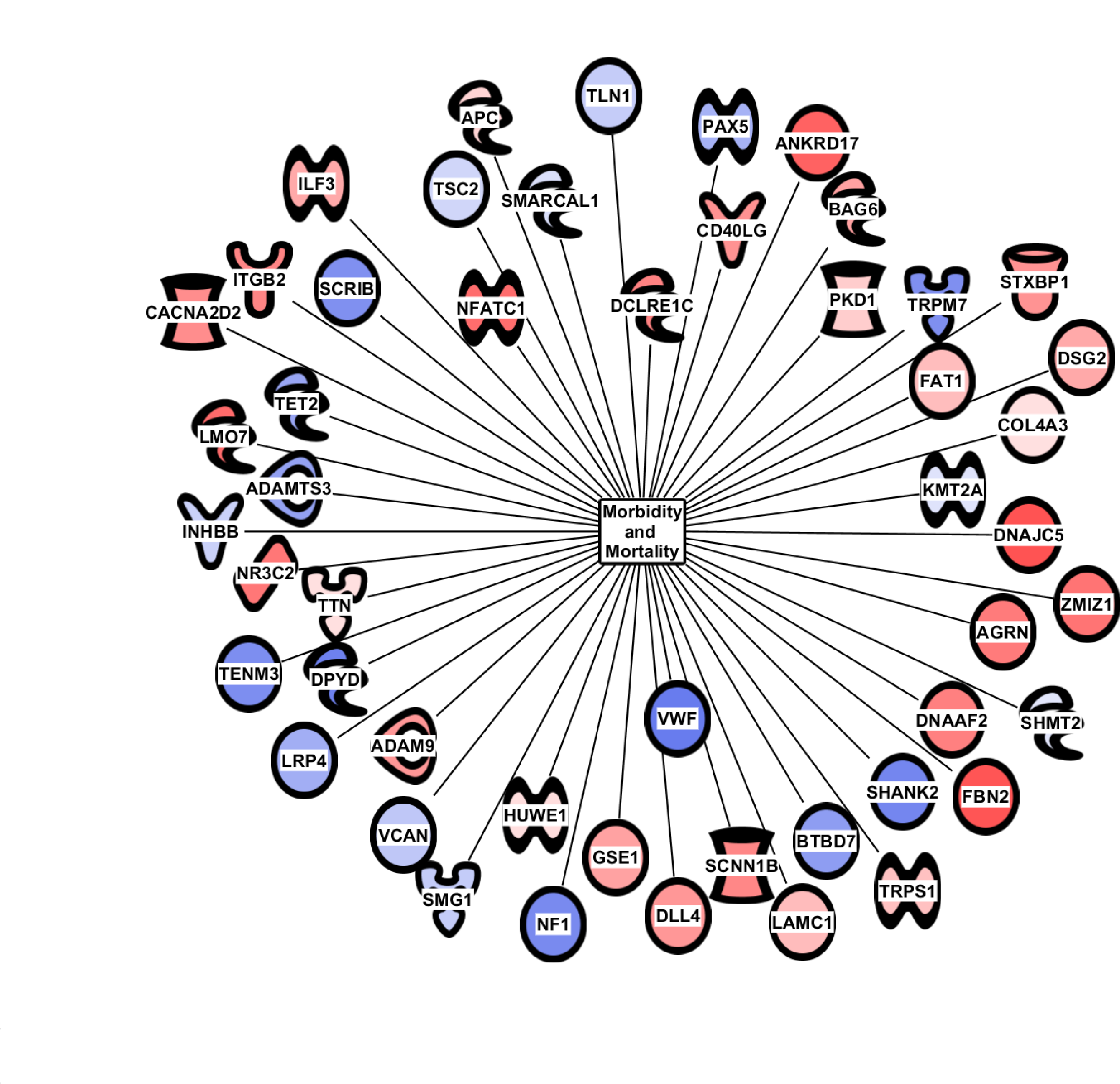

Supplement: S2 Fig — (TIF) [file pone.0234539.s003.tif]
